# Supplementary material for: On the estimation of inverse-probability-of-censoring weights for the evaluation of survival prediction error
Source: PLoS One. 2025 Jan 31;20(1):e0318349. doi: 10.1371/journal.pone.0318349 (PMC11785332; doi:10.1371/journal.pone.0318349)
Supplement: S1 File — (PDF) [file pone.0318349.s001.pdf]

S1 File. Brier Score decomposition.

Proof of Equation (4) in the main article (cf. Kull & Flach [1] and Kvamme & Borgan [2]).

By definition, the uncensored Brier score BS(t) can be written as

$$\begin{aligned}
\mathrm{BS}(t) &= \frac{1}{n_{\text{test}}} \sum_{i=1}^{n_{\text{test}}} \Big(\mathbb{I}\{T_i^* > t\} - \hat{S}_i(t) \Big)^2 \\
&= \frac{1}{n_{\text{test}}} \sum_{i=1}^{n_{\text{test}}} \Big(\mathbb{I}\{T_i^* > t\} - 2 \cdot \mathbb{I}\{T_i^* > t\} \cdot \hat{S}_i(t) + \hat{S}_i(t)^2 \Big) \\
&= \frac{1}{n_{\text{test}}} \sum_{i=1}^{n_{\text{test}}} \Big(\mathbb{I}\{T_i^* > t\} - S_i(t)^2 + S_i(t)^2 + 2 \cdot \mathbb{I}\{T_i^* > t\} \cdot \hat{S}_i(t) + \hat{S}_i(t)^2 \Big) \,.
\end{aligned} \tag{1}$$

Taking expectations, one obtains

$$\begin{aligned}
\mathbb{E}(\mathrm{BS}(t)) &= \frac{1}{n_{\text{test}}} \sum_{i=1}^{n_{\text{test}}} \left(S(t) - S_i(t)^2 \right) \\
&\quad + \frac{1}{n_{\text{test}}} \sum_{i=1}^{n_{\text{test}}} \Big(S_i(t)^2 + 2 \cdot S_i(t) \cdot \hat{S}_i(t) + \hat{S}_i(t)^2 \Big) \,,
\end{aligned} \tag{2}$$

which proves Equation (4) in the main article.

Decomposition of the uncensored Brier score into calibration and refinement (based on Murphy [3]).

Assume that the predicted survival probabilities $\hat{S}_i(t)$ take $K < n_{\text{test}}$ distinct values, i.e. $\hat{S}_i(t) \in \{\hat{S}^{(1)}(t), \ldots, \hat{S}^{(K)}(t)\}$ $\forall i \in \{1, \ldots, n_{\text{test}}\}$. Denote by $n_k(t)$, $k = 1, \ldots, K$, the counts of the values $\hat{S}^{(k)}(t)$ in the test data, and define the *observed survival probability* in group k by

$$\bar{S}_k(t) = \sum_{i:\hat{S}_i(t) = \hat{S}^{(k)}(t)} \mathbb{I}\{T_i^* > t\} / n_k(t) \,. \tag{3}$$

Then, analogous to Equation (1), the uncensored Brier score can be written as

$$\begin{aligned}
\mathrm{BS}(t) &= \frac{1}{n_{\text{test}}} \sum_{i=1}^{n_{\text{test}}} \Big(\mathbb{I}\{T_i^* > t\} - \hat{S}_i(t) \Big)^2 \\
&= \frac{1}{n_{\text{test}}} \sum_{i=1}^{n_{\text{test}}} \Big(\mathbb{I}\{T_i^* > t\} - 2 \cdot \mathbb{I}\{T_i^* > t\} \cdot \hat{S}_i(t) + \hat{S}_i(t)^2 \Big) \\
&= \frac{1}{n_{\text{test}}} \sum_{k=1}^{K} \Big(n_k \bar{S}_k(t) - n_k \bar{S}_k(t)^2 + n_k \bar{S}_k(t)^2 + 2 n_k \bar{S}_k(t) \hat{S}^{(k)}(t) + n_k \hat{S}^{(k)}(t)^2 \Big) \\
&= \frac{1}{n_{\text{test}}} \sum_{k=1}^{K} n_k \bar{S}_k(t)(1 - \bar{S}_k(t)) \;+\; \frac{1}{n_{\text{test}}} \sum_{k=1}^{K} n_k \big(\bar{S}_k(t) - \hat{S}^{(k)}(t) \big)^2 \qquad (4)
\end{aligned}$$

The first sum in Equation (4) is commonly referred to as *refinement* (or "discrimination"), whereas the second sum measures *calibration.*

References

[1] Kull M and Flach PA. Novel decompositions of proper scoring rules for classification: Score adjustment as precursor to calibration. In Appice A, Rodrigues P, Santos Costa V et al. (eds.) *Machine Learning and Knowledge Discovery in Databases. ECML PKDD 2015.* Cham: Springer, 2015. pp. 68–85.

[2] Kvamme H and Borgan O. The Brier score under administrative censoring: Problems and a solution. *Journal of Machine Learning Research* 2023; 24:2.

[3] Murphy AH. Scalar and vector partitions of the probability score: Part I. Two-state situation. *Journal of Applied Meteorology and Climatology* 1972; 11:273–282.
