# Supplementary material for: On the estimation of inverse-probability-of-censoring weights for the evaluation of survival prediction error
Source: PLoS One. 2025 Jan 31;20(1):e0318349. doi: 10.1371/journal.pone.0318349 (PMC11785332; doi:10.1371/journal.pone.0318349)
Supplement: S2 File — (PDF) [file pone.0318349.s002.pdf]

S2 File. Tuning details.

In this section we detail the tuning procedures used for the Lasso, random forest and XGBoost models in the simulation study and the SEER application. The Lasso models were fitted using the `cv.glmnet` function provided in the R package **glmnet** [1], which uses k-fold cross-validation to determine the optimal regularization parameter λ. The number of folds k was set to 10 (the default). The estimated regression coefficients of the model with the lowest mean cross-validated error (`cvm`), provided in the `lambda.min` object, were input into a user defined function (`predict.glmnet`) to calculate the survival and censoring survival function using Breslow's method. The random forest and XGBoost models were tuned using Bayesian optimization, implemented in the R package **parBayesianOptimization** [2]. This method explores the parameter space by fitting Gaussian processes to any target function that can be maximized, e.g., the negative mean squared error or the area under the curve, and subsequently maximizing utility functions reflecting the "usefulness" of exploring previously unexplored space. This allows for a targeted search of the part of the parameter space where the global maximum of the target function is most likely to be located, thus rendering the search more efficient, as compared to, e.g., a grid-based search. We chose to maximize the negative out-of-bag prediction error for random forest, obtained from the `ranger` function from the eponymous R package [3], and the log-likelihood for XGBoost, obtained from the `xgb.cv` function implemented in the **xgboost** package [4], with $k = 5$ folds and $nrounds = 1000$. The utility function used was the `upper confidence bound` (`ucb`, the default). The search is initialized by setting the bounds of the space of hyperparameter values to be searched and ends after a pre-specified number of optimization iterations. In random forest, we tuned the following hyperparameters, with the upper and lower bounds of the space to be searched in parentheses: `mtry` (1, $p + q$, i.e., the total number of predictors), `max.depth` (1, 10), `min.node.size` (1, 10), and `sample.fraction` (0.1, 1). In XGBoost, we

tuned the following hyperparameters: `eta` (0.01, 0.3), `max_depth` (1, 10), `min_child_weight` (1, 100), `subsample` (0.1, 1), `colsample_bytree` (0.1, 1), `gamma` (0, 10), `lambda` (1, 10), and `alpha` (1, 10). We limited the number of optimization steps (`iters.n`) to a maximum of 8 for both random forest and XGBoost.

References

[1] Friedman J, Tibshirani R and Hastie T. Regularization paths for generalized linear models via coordinate descent. *Journal of Statistical Software* 2010; 33:1.

[2] Wilson S. *ParBayesianOptimization: Parallel Bayesian Optimization of Hyperparameters*, 2022. R package version 1.2.6.

[3] Wright MN and Ziegler A. ranger: A fast implementation of random forests for high dimensional data in C++ and R. *Journal of Statistical Software* 2017; 77:1.

[4] Chen T and Guestrin C. xgboost: A scalable tree boosting system. In *Proceedings of the 22nd ACM SIGKDD International Conference on Knowledge Discovery and Data Mining*. New York, NY, USA: Association for Computing Machinery, pp. 785–794.
