# Supplementary material for: On the estimation of inverse-probability-of-censoring weights for the evaluation of survival prediction error
Source: PLoS One. 2025 Jan 31;20(1):e0318349. doi: 10.1371/journal.pone.0318349 (PMC11785332; doi:10.1371/journal.pone.0318349)
Supplement: S3 File — (PDF) [file pone.0318349.s003.pdf]

S5 File. SEER application: semi-synthetic analysis.

Methods

In order to complement our analysis of the original SEER data, we performed additional analyses using a semi-synthetic version of the SEER dataset. This semi-synthetic dataset was obtained following the procedure proposed by Qi et al. [1]. The preprocessing steps for the SEER data are described in the main article. Additionally, for the semi-synthetic analysis, we simplified the preprocessed SEER data by dropping two additional variables (order of radiation and surgery, adjusted TNM status) and by recoding three additional variables (marital status, race, type of radiation therapy) to include fewer classes. This was done in order to ensure stable model fits, since these variables were found to be highly unbalanced. We then performed stratified synthetic censoring of the preprocessed SEER dataset. For this we proceeded in three steps. First, we estimated the censoring distribution separately for each of the two groups of observations defined by the binary ER status variable using a marginal Kaplan-Meier model without covariates. We chose to stratify by ER status because we found that the estimated censoring distributions were different in the two groups and because this variable was relatively balanced. Second, we removed all of the censored observations from the preprocessed SEER dataset ($n = 121,798$), leaving only the uncensored observations (n = 8,400). Finally, we artificially censored the remaining observations by sampling censoring times from the censoring distributions estimated during the first step and applying these to the uncensored observations. This resulted in a censoring rate of 35.4% for the 8,400 observations. Using this semi-synthetic dataset we then performed the same analyses as with the non-synthetic dataset.

Results

The following results provide an analysis of the predictive performance of three estimation methods—Cox proportional hazards, random forest and XGBoost—as a function of time and of the estimated censoring survival function using the IPCW Brier score on the semi-synthetic dataset. In the case of the Cox proportional hazards model, the predictive performance does not seem to be severely impacted by the choice of the data used to fit the censoring function. In the case of the machine learning models, the censoring models fit to the test set seem to slightly outperform the models fit to either the training dataset or the combined dataset. This is especially evident for the models fit using XGBoost. These differences could be attributable to the fact that all of the models are evaluated using the test data, which confers an advantage to the models that were also fit using that dataset. Machine learning models in particular have a high tendency to overfit the data used during the training process. Additionally, unlike in the simulation study, where we had access to the "whole" ground truth (i.e., the true survival and censoring survival functions), here we only know the censoring survival function. Hence, in this setting, it is difficult to distinguish between real differences between the models and differences due to inherent biases in the modeling pipeline.

Another interesting finding, which is in line with the results of the analysis of the non-synthetic SEER dataset (see the main article), is the importance of prior tuning of the ML models. Figs 2 and 3 show the prediction error curves for the models fit using random forest and XGBoost, for both the untuned models (with the hyperparameters set to the default values pre-specified in the respective R packages, see Section S2), as well as for the models where the most important hyperparameters were tuned. Both XGBoost as well as random forest clearly benefit from prior tuning and exhibit a sharp decrease in the Brier score values when tuned.

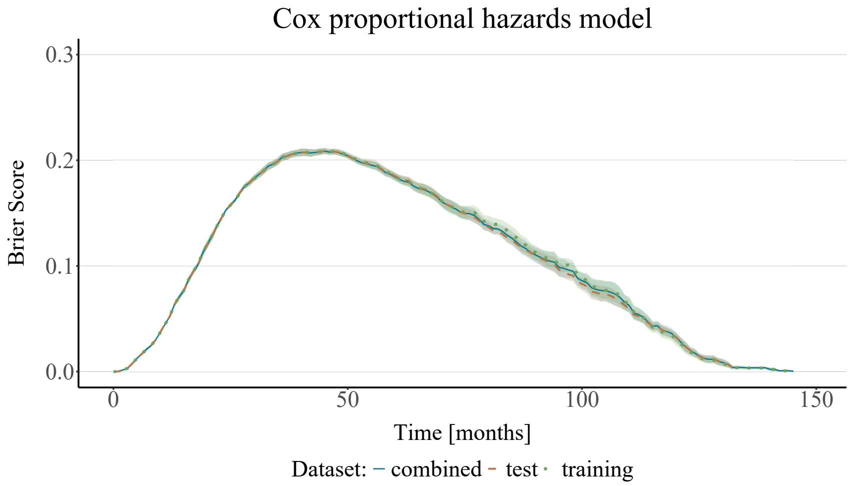

Figure 1: Analysis of the semi-synthetic SEER breast cancer data using a Cox proportional hazards model. The plot shows the mean (bold center line) and standard deviation (shaded area) of the IPCW Brier score obtained on 10 bootstrap test samples, with IPC weights estimated from either the training, test, or the combined dataset. A Cox proportional hazards model was used for estimating the survival and the censoring survival functions.

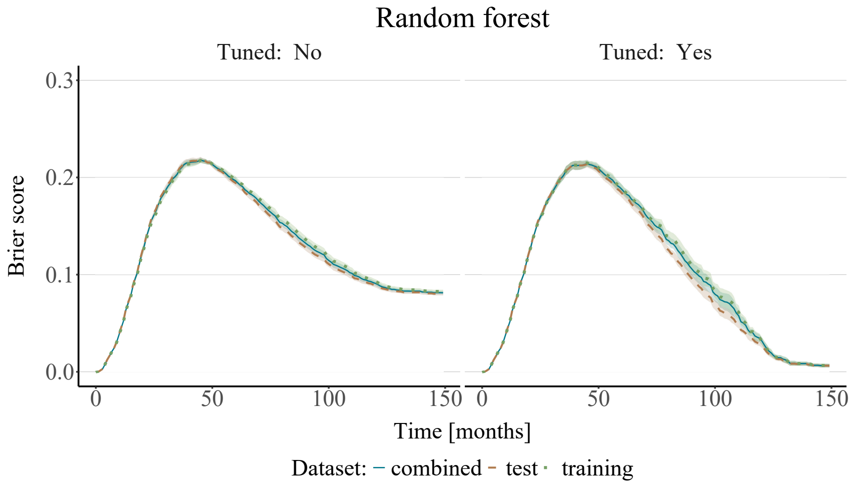

Figure 2: Analysis of the semi-synthetic SEER breast cancer data using random forest. The plots show the mean (bold center line) and standard deviation (shaded area) of the IPCW Brier score obtained on 10 bootstrap test samples, with IPC weights estimated from either the training, test, or the combined dataset. A random forest was used for estimating the survival and censoring survival functions. The left panel shows the results of an untuned model, with the number of trees set to 500 and all other hyperparameters set to their default values, whereas the model in the right panel was tuned using Bayesian optimization.

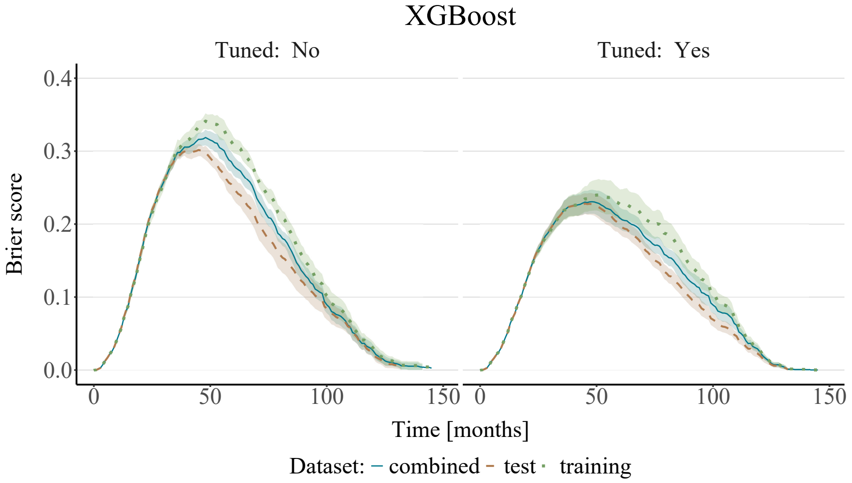

Figure 3: Analysis of the semi-synthetic SEER breast cancer data using XGBoost. The plots show the mean (bold center line) and standard deviation (shaded area) of the IPCW Brier score obtained on 10 bootstrap test samples, with IPC weights estimated from either the training, test, or the combined dataset. XGBoost was used for estimating the survival and censoring survival functions. The left panel shows the results of an untuned model, with the number of boosting rounds set to 500 and all other hyperparameters set to their default values, whereas the model on the right was tuned using cross-validation and Bayesian optimization.

References

[1] Qi AS, Kumar N, Farrokh M, Sun W, Kuan LH, Ranganath R, Henao R and Greiner R. An effective meaningful way to evaluate survival models. In *Proceedings of the 40th International Conference on Machine Learning* 2023; 202: 28244-28276.
