# Supplementary material for: On the estimation of inverse-probability-of-censoring weights for the evaluation of survival prediction error
Source: PLoS One. 2025 Jan 31;20(1):e0318349. doi: 10.1371/journal.pone.0318349 (PMC11785332; doi:10.1371/journal.pone.0318349)
Supplement: S1 Table — (PDF) [file pone.0318349.s004.pdf]

S3 Tables. Simulation: additional results.

In this section we detail some of the numerical results of the simulation study for scenarios (i) through (v). The tables below show the empirical median and interquartile range (IQR) of the root weighted squared error (RWSE).

Table 1: Numerical results for the baseline scenario (i) with varying n.

n	dataset	median	IQR
50	combined	0.122298	0.092393
	test	0.122027	0.092023
	training	0.121727	0.093373
100	combined	0.052218	0.026807
	test	0.051732	0.027178
	training	0.052262	0.026556
200	combined	0.030809	0.014135
	test	0.030563	0.013782
	training	0.031133	0.013724
500	combined	0.016770	0.006046
	test	0.016703	0.005960
	training	0.017155	0.006220
1000	combined	0.012272	0.004449
	test	0.012217	0.004377
	training	0.012466	0.004449

Table 2: Numerical results for the baseline scenario (i) with varying n_{training}.

n_{training}	dataset	median	IQR
25	combined	0.092088	0.090599
	test	0.092028	0.090606
	training	0.090900	0.094552
50	combined	0.029990	0.024008
	test	0.030003	0.024146
	training	0.030277	0.024051
100	combined	0.016783	0.008631
	test	0.016673	0.008607
	training	0.017406	0.008743
250	combined	0.012705	0.004391
	test	0.012599	0.004378
	training	0.012987	0.004606
500	combined	0.012172	0.004353
	test	0.012179	0.004285
	training	0.012454	0.004545

Table 3: Numerical results for the baseline scenario (i) with varying $n_{\mathbf{test}}$.

n_{test}	dataset	median	IQR
25	combined	0.048362	0.018464
	test	0.046727	0.018721
	training	0.048398	0.018653
50	combined	0.035430	0.012966
	test	0.034604	0.012976
	training	0.035453	0.013006
100	combined	0.024863	0.009574
	test	0.024559	0.009277
	training	0.024935	0.009656
250	combined	0.016801	0.005737
	test	0.016437	0.005889
	training	0.017012	0.005862
500	combined	0.012218	0.004472
	test	0.012140	0.004221
	training	0.012492	0.004563

Table 4: Numerical results for the baseline scenario (i) with varying censoring rate.

censoring rate	dataset	median	IQR
0.2	combined	0.008502	0.003464
	test	0.008523	0.003410
	training	0.008678	0.003430
0.5	combined	0.012214	0.004461
	test	0.012208	0.004204
	training	0.012485	0.004514
0.8	combined	0.033016	0.010840
	test	0.032862	0.010817
	training	0.032989	0.010657

Table 5: Numerical results for the Weibull-Cox scenario (ii) with varying n.

n	dataset	median	IQR
50	combined	0.105970	0.079343
	test	0.105782	0.080984
	training	0.120051	0.089729
100	combined	0.048698	0.023401
	test	0.046920	0.023029
	training	0.052495	0.026344
200	combined	0.028000	0.012002
	test	0.027209	0.011742
	training	0.029429	0.013374
500	combined	0.015746	0.005491
	test	0.015171	0.005454
	training	0.016540	0.005897
1000	combined	0.010777	0.003884
	test	0.010589	0.003836
	training	0.011306	0.004032

Table 6: Numerical results for the Weibull-Cox scenario (ii) with varying n_{training}.

n_{training}	dataset	median	IQR
25	combined	0.070954	0.078706
	test	0.071016	0.079037
	training	0.082263	0.083034
50	combined	0.025268	0.019958
	test	0.025207	0.019793
	training	0.029391	0.020515
100	combined	0.014135	0.006967
	test	0.014065	0.006970
	training	0.015830	0.007604
250	combined	0.011305	0.003856
	test	0.011133	0.003875
	training	0.012033	0.004296
500	combined	0.010875	0.003837
	test	0.010676	0.003719
	training	0.011306	0.003989

Table 7: Numerical results for the Weibull-Cox scenario (ii) with varying n_{test}.

n_{test}	dataset	median	IQR
25	combined	0.046778	0.019038
	test	0.042462	0.017879
	training	0.047463	0.019086
50	combined	0.034291	0.012707
	test	0.031319	0.011458
	training	0.034693	0.013006
100	combined	0.024348	0.008548
	test	0.022965	0.008244
	training	0.024733	0.009006
250	combined	0.015481	0.005717
	test	0.014883	0.005604
	training	0.015919	0.005833
500	combined	0.010799	0.003827
	test	0.010605	0.003724
	training	0.011347	0.004024

Table 8: Numerical results for the Weibull-Cox scenario (ii) with varying censoring rate.

censoring rate	dataset	median	IQR
0.2	combined	0.008619	0.003138
	test	0.008468	0.003179
	training	0.009055	0.003452
0.5	combined	0.010812	0.003800
	test	0.010595	0.003628
	training	0.011266	0.003970
0.8	combined	0.019130	0.007838
	test	0.019244	0.007836
	training	0.019375	0.008027

Table 9: **Numerical results for the misspecification scenario (iii).**

n	dataset	estimator	median	IQR
50	combined	Cox	0.105096	0.075661
		KM	0.115674	0.080021
	test	Cox	0.104620	0.076449
		KM	0.115508	0.078604
	training	Cox	0.120273	0.088433
		KM	0.113973	0.081371
100	combined	Cox	0.048076	0.023708
		KM	0.052856	0.027739
	test	Cox	0.046759	0.022437
		KM	0.052522	0.027285
	training	Cox	0.052203	0.028104
		KM	0.052736	0.027447
200	combined	Cox	0.028219	0.011927
		KM	0.031765	0.014400
	test	Cox	0.027414	0.011541
		KM	0.031680	0.014480
	training	Cox	0.029521	0.013195
		KM	0.031706	0.014722
500	combined	Cox	0.015695	0.005643
		KM	0.020745	0.008104
	test	Cox	0.015191	0.005494
		KM	0.020648	0.007757
	training	Cox	0.016437	0.005893
		KM	0.020788	0.008476
1000	combined	Cox	0.010709	0.003828
		KM	0.017166	0.006058
	test	Cox	0.010571	0.003720
		KM	0.017053	0.005995
	training	Cox	0.011238	0.003934
		KM	0.017129	0.006211

Table 10: Numerical results for the low-noise scenario (iv).

estimator	dataset	median	IQR
Cox	training	0.011343	0.004063
	test	0.010676	0.003676
	combined	0.010844	0.003839
Lasso	training	0.061465	0.015294
	test	0.061208	0.015224
	combined	0.062695	0.015285
Random forest	training	0.018313	0.008575
	test	0.015233	0.007669
	combined	0.016479	0.008632
XGBoost	training	0.126976	0.017043
	test	0.129024	0.017155
	combined	0.129084	0.017216

Table 11: Numerical results for the high-noise scenario (v).

estimator	dataset	median	IQR
Cox	training	0.014445	0.006019
	test	0.012807	0.005311
	combined	0.013351	0.005713
Lasso	training	0.039958	0.015127
	test	0.040777	0.014950
	combined	0.042597	0.015658
Random forest	training	0.025507	0.009724
	test	0.017430	0.007963
	combined	0.019180	0.008871
XGBoost	training	0.028196	0.012078
	test	0.022956	0.010475
	combined	0.021264	0.010226
