# Supplementary material for: On the estimation of inverse-probability-of-censoring weights for the evaluation of survival prediction error
Source: PLoS One. 2025 Jan 31;20(1):e0318349. doi: 10.1371/journal.pone.0318349 (PMC11785332; doi:10.1371/journal.pone.0318349)
Supplement: S1 Fig — (PDF) [file pone.0318349.s005.pdf]

S4 Figs. SEER application: additional figures.

This section contains some additional figures from the analysis of the SEER data, with the shaded bands representing the standard deviation removed to improve legibility (see Figs 6 – 8 in the main article).

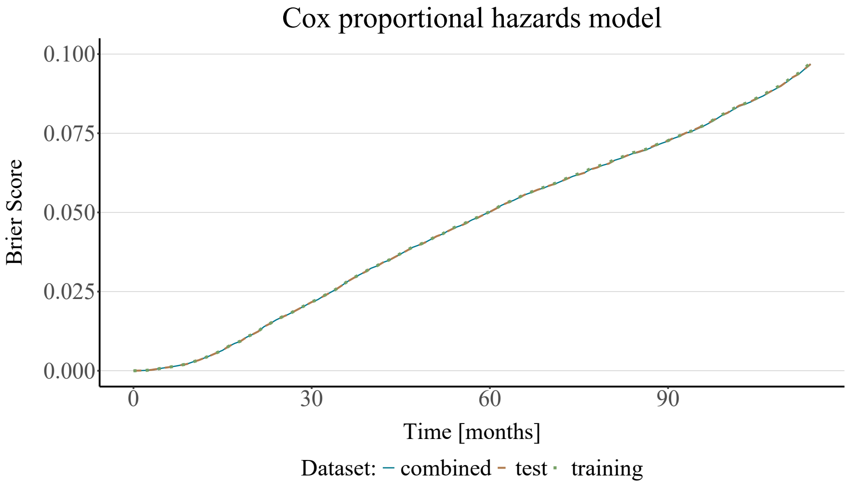

Figure 1: Analysis of the SEER breast cancer data using a Cox proportional hazards model. The plot shows the mean values of the IPCW Brier score obtained on 10 bootstrap test samples, with IPC weights estimated from either the training, test, or the combined dataset. A Cox proportional hazards model was used for estimating the survival and the censoring survival functions.

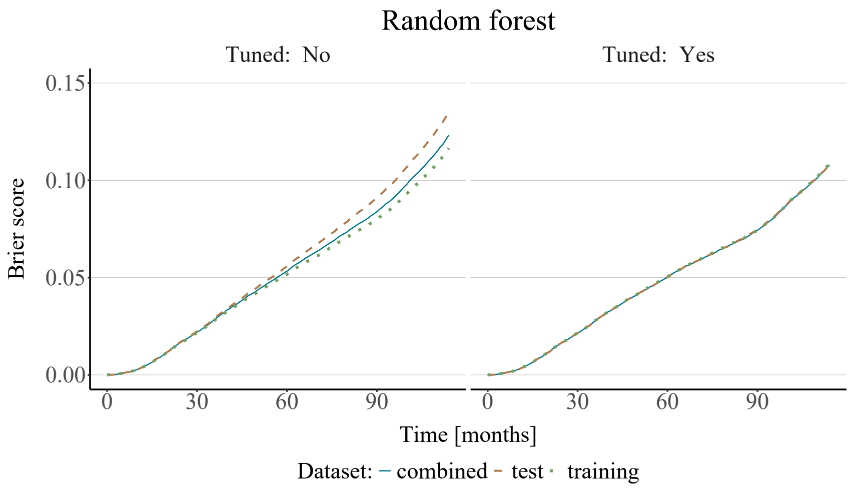

Figure 2: Analysis of the SEER breast cancer data using random forest. The plots show the mean values of the IPCW Brier score obtained on 10 bootstrap test samples, with IPC weights estimated from either the training, test, or the combined dataset. A random forest was used for estimating the survival and censoring survival functions. The left panel shows the results of an untuned model, with the number of trees set to 500 and all other hyperparameters set to their default values, whereas the model in the right panel was tuned using Bayesian optimization.

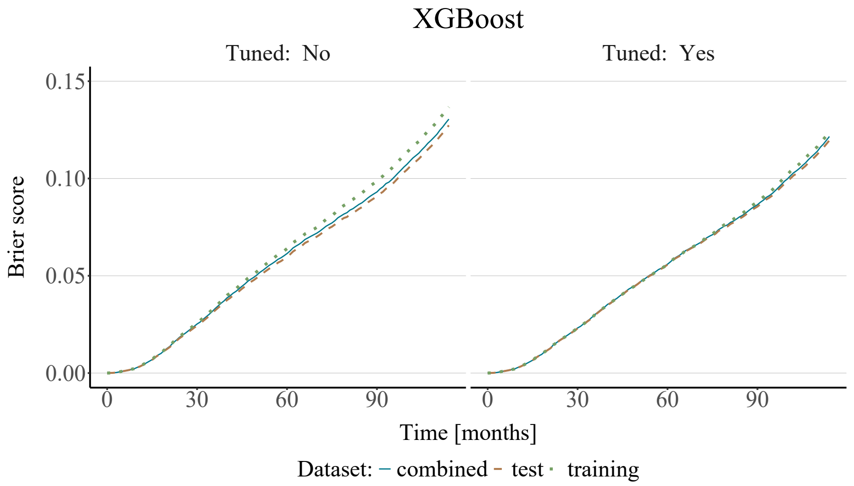

Figure 3: Analysis of the SEER breast cancer data using XGBoost. The plots show the mean values of the IPCW Brier score obtained on 10 bootstrap test samples, with IPC weights estimated from either the training, test, or the combined dataset. XGBoost was used for estimating the survival and censoring survival functions. The left panel shows the results of an untuned model, with the number of boosting rounds set to 500 and all other hyperparameters set to their default values, whereas the model on the right was tuned using cross-validation and Bayesian optimization.
