# Supplementary material for: On the estimation of inverse-probability-of-censoring weights for the evaluation of survival prediction error
Source: PLoS One. 2025 Jan 31;20(1):e0318349. doi: 10.1371/journal.pone.0318349 (PMC11785332; doi:10.1371/journal.pone.0318349)
Supplement: Attachment — Submitted filename: Response to Reviewers.pdf [file pone.0318349.s006.pdf]

Revision of PONE-D-24-45479 – Point-by-Point Reply to the Academic Editor's and the Reviewer's Comments

We thank the academic editor and the reviewer for evaluating our work and for very helpful comments and suggestions. According to their recommendations, we have prepared a revised version of the paper. Major changes to the manuscript have been marked red. Detailed point-by-point replies to the academic editor's and the reviewer's comments are given below.

Reply to the Academic Editor:

Thank you very much for submitting this manuscript to the journal. Our referee has reviewed your manuscript and she/he provides positive feedback for your work. I would like to invite you to prepare a revised manuscript and re-submit the revision to the journal.

We thank the academic editor for his positive feedback and for inviting us to prepare and re-submit a revised manuscript to PLOS ONE.

In addition to the referee's comment, I also recall that the published paper "Chen, L.-P. (2020). Causal inference for left-truncated and right-censored data with covariates measurement error. Computational & Applied Mathematics, 39:126. DOI: 10.1007/s40314-020-01152-4." also adopts the inverse-probability-of-censoring weights for the average treatment effect estimation in causal inference. I would suggest the authors to cite this reference to enhance the comprehension of literature review.

Following the suggestion of the academic editor, we have referenced the paper by Chen et al. in the discussion (Section 5) of the revised paper (please see p. 21).

Reply to Reviewer 1:

This paper addresses an interesting question in the estimation paradigm for the IPCW-based Brier score. A disagreement exists in the literature regarding the optimal approach for splitting the dataset to estimate the censoring distribution. This study makes a meaningful contribution by using various configurations of synthetic datasets alongside the SEER breast cancer dataset to empirically identify the best strategy for model evaluation.

The authors have thoughtfully investigated an overlooked issue in the community. Although the findings align with intuition and are not groundbreaking, the work is rigorous and offers significant value. The manuscript is well-organized, with clear

motivation for the research questions, and the authors provide a solid overview of the Brier score and IPCW weighting methods. The experimental design is thorough, addressing a range of scenarios and adding to the study's robustness.

Thank you very much for these favorable statements and for your positive evaluation of our work.

While the paper is largely ready for publication, there are a few optional enhancements that could further strengthen the manuscript. However, these are good to have, not a must:

1. Currently, the synthetic experiments compare the IPCW Brier score with the expected Brier (Eq 4). Adding an additional comparison with the mean squared error (Eq 2) might provide a more comprehensive perspective.

We agree with the reviewer that investigating the mean squared error is a key issue in the evaluation of survival predictions. In fact, our approach closely follows this rationale: In our comparisons, we chose the expected uncensored Brier Score (Eq 4, a theoretical quantity incorprating the squared difference between observed and predicted survival probabilities) as the reference score (= ground-truth). Our choice was motivated by the fact that the IPCW Brier Score (Eq 5, our estimator of interest) is an asymptotically unbiased approximation of the uncensored Brier Score.

On the other hand, the uncensored Brier Score (and thus also its estimator, the IPCW Brier Score) is a *biased* approximation of the mean squared error, with the bias depending on the unknown true survival function (see Eq 4). Consequently, and since this bias is the same for all censoring estimators investigated in our paper, a comparison of the IPCW Brier score to the mean squared error would not have led to any changes in the conclusions drawn from our simulations. Furthermore, we were unable to compute the bias (it being entirely dependent on unknown quantities) in our analysis of the SEER data. In view of these considerations, we feel that a comparison of the IPCW Brier score to the uncensored Brier score is the best we can do to perform an unbiased squared-error-type IPCW analysis.

2. The current synthetic datasets are designed by heuristics, which means they can hardly represent the distributions in the real-world. Qi et al. [1] proposed a way to generate realistic semi-synthetic survival datasets which contains real-world features, real-world event distribution, and close-to-real-world censor distribution. Implementing experiments with these semi-synthetic datasets would further enhance the study's applicability.

Thank you for making us aware of this very interesting approach. Following

the reviewer's suggestion, we extended our empirical study in Section 4 by the analysis of a semi-synthetic data set. More specifically, we applied the method by Qi et al. to the subsample of uncensored observations in the SEER breast cancer data ($n = 8{,}400$), generating additional censoring times from a stratified Kaplan-Meier estimate of the original censoring distributions. The results obtained from our semi-synthetic analysis essentially confirm the results obtained from the original SEER data, please see the supporting information of the revised paper (Section S5).

3. For completeness, a brief overview of the proofs for (1) the decomposition of the Brier score into discrimination and calibration components, and (2) the expected Brier score's decomposition into the MSE and variance terms, would be valuable additions. Such can be done in the supplementary.

Following the reviewer's suggestion, we included the proofs of Eq 4 (decomposition of the Brier Score into the MSE and a bias component) and of the decomposition of the Brier Score into calibration and refinement components in the supporting information of the paper (Section S1), following Murphy [1], Kull & Flach [2] and Kvamme & Borgan [3].

4. The conclusion discusses the concordance index as an alternative evaluation metric. Expanding this section to include other metrics, such as the mean absolute or squared error between predicted and actual event times, as suggested in [1], would provide a more comprehensive evaluation framework.

Following the reviewer's suggestion, we added a brief discussion of alternative evaluation metrics such as the mean absolute error (MAE) to the discussion (Section 5, please see pp. 23–24).

References

[1] Murphy AH. Scalar and vector partitions of the probability score: Part I. Two-state situation. *Journal of Applied Meteorology and Climatology* 1972; 11:273–282.

[2] Kull M and Flach PA. Novel decompositions of proper scoring rules for classification: Score adjustment as precursor to calibration. In Appice A, Rodrigues P, Santos Costa V et al. (eds.) *Machine Learning and Knowledge Discovery in Databases. ECML PKDD 2015.* Cham: Springer, 2015. pp. 68–85.

[3] Kvamme H and Borgan O. The Brier score under administrative censoring: Problems and a solution. *Journal of Machine Learning Research* 2023; 24:2.
